# Incidental Detection of Acute Leukemia During Genetic Evaluation of Neurodevelopmental Disorder in a Pediatric Clinic: A Case Report

**DOI:** 10.1155/crpe/1527918

**Published:** 2026-07-15

**Authors:** Stephen G. Jones

**Affiliations:** ^1^ Department of Pediatrics, University of Arkansas for Medical Sciences, Little Rock, Arkansas, USA, uams.edu

**Keywords:** acute leukemia, autism spectrum disorder, case report, chromosomal microarray, hypodiploidy, incidental finding

## Abstract

A previously healthy 5‐year‐old boy presented to pediatric neurology for evaluation of recurrent migraine headaches with associated anxiety. Neurologic imaging was unremarkable, but clinical features consistent with autism spectrum disorder were noted. As part of standard‐of‐care ASD evaluation, SNP chromosomal microarray (CMA) on peripheral blood revealed a hypodiploid genome (∼35 chromosomes), suspicious for leukemia. Prompt notification led to repeat blood testing showing ∼30% circulating blasts. A diagnosis of acute leukemia was made, and constitutional CMA on buccal DNA showed a normal diploid male karyotype. This case illustrates the potential for ASD genetic testing to yield critical, incidental systemic findings and underscores the importance of rapid follow‐up and interdisciplinary care.

## 1. Introduction

Autism spectrum disorder (ASD) is a neurodevelopmental condition with diverse clinical presentations and heterogeneous genetic underpinnings. Chromosomal microarray analysis (CMA) is a relatively simple first‐tier test in the genetic evaluation of ASD and is recommended as the initial genetic test for individuals with developmental delays or congenital anomalies [1, 2]. While the primary goal of CMA is to identify pathogenic copy number variants or uniparental disomy relevant to developmental disorders, incidental findings may emerge. Here, we report a unique case in which SNP‐based CMA, performed as part of a neurologic workup for ASD, revealed evidence of a hypodiploid genome, leading to the unexpected diagnosis of acute leukemia in an otherwise asymptomatic child.

## 2. Case Presentation

A previously healthy 5‐year‐old male was referred to the pediatric neurology clinic for evaluation of recurrent headaches. The headache episodes were consistent with migraine by character and description, without red flag features, and were notably exacerbated by emotional stress. MRI of the brain without contrast was unremarkable. Of note, there was no report or complaint of excessive fatigue, frequent infection, or easy bleeding or bruising, and the patient appeared to otherwise be in a good state of health.

During the neurologic evaluation, clinical features consistent with ASD were observed, including limited social reciprocity, restricted interests, and communication differences. In line with best practice guidelines for genetic evaluation in ASD, SNP‐based CMA was performed on peripheral blood.

The CMA report identified an incidental finding suggestive of a hypodiploid genome, with evidence of a reduced chromosome count consistent with approximately 35 chromosomes. The hypodiploid pattern identified on peripheral blood CMA was concerning for a somatic leukemic clone rather than constitutional chromosomal hypodiploidy. The patient’s primary care pediatrician and neurology team were promptly notified of this finding on the day of their discovery.

As per the recommendation from the reviewing pathologist, repeat peripheral blood testing was obtained and evaluated by an outside diagnostic laboratory within a week. Manual differential and pathologist review of the peripheral smear revealed approximately 30% circulating lymphoblasts, concerning for acute lymphoblastic leukemia (ALL). The patient was urgently referred to pediatric hematology/oncology and was able to be seen 6 days later in their clinic. Upon prompt evaluation, a diagnosis of ALL was confirmed.

The patient’s family elected to pursue treatment for his newly diagnosed leukemia at a local hospital. To confirm that the hypodiploid pattern seen on CMA was not constitutional, a follow‐up CMA was performed on buccal swab DNA, which returned normal male results: arr(1–22)x2,(X,Y)x1.

## 3. Discussion

This case represents a rare but critical example of an incidental diagnosis of acute leukemia made through genetic testing intended for neurodevelopmental assessment. Hypodiploidy, defined as a chromosome count below the normal diploid number, is a cytogenetic feature that, while rare in constitutional DNA, is strongly associated with certain hematologic malignancies, especially ALL. Hypodiploid ALL is known to carry a poorer prognosis and often requires aggressive therapy [3].

Subclassification of hypodiploid ALL into near‐haploid (24–31 chromosomes) and low‐hypodiploid (32–39 chromosomes) subtypes is essential, as the presence of a doubled clone can mimic hyperdiploidy and delay recognition of high‐risk disease [4]. Hypodiploid ALL has been associated with mutations in TP53 and other genes related to genomic instability, guiding risk stratification and therapy, often including hematopoietic stem cell transplant in first remission [5]. Clonal hematologic malignancies may harbor somatic chromosomal anomalies or evolve from a genetically distinct subpopulation, and these clonal dynamics are well described in relapsed ALL [6].

SNP‐based CMA platforms can detect genome‐wide copy number changes, regions of copy‐neutral loss of heterozygosity, mosaicism, and aneuploidy. When peripheral blood is used as the DNA source, the presence of a sufficiently large leukemic clone may produce abnormal chromosomal patterns reflective of malignant hematopoietic cells rather than constitutional DNA [7, 8]. In this way, SNP‐CMA performed for neurodevelopmental evaluation may inadvertently identify clonal hematologic abnormalities, including leukemia‐associated hypodiploidy. While this is not the primary intent of such testing, clinicians must remain vigilant to atypical results that could signal broader systemic disease.

Although the unique scenario of our patient was not encountered through review of available literature, roughly 6% of children with leukemia are asymptomatic at diagnosis [9]. These discoveries can be made through routine screening or incidentally such as through preoperative labs or through an emergency department visit for another reason. As the prevalence of autism has been reported to be approximately 1.7% and increasing in recent years [10], the potential for a similar incidental detection certainly exists.

This case further underscores the importance of close interdepartmental communication, including pediatrics, pathology, neurology, genetics, and oncology, when abnormal laboratory findings may represent critical systemic disease. In our case, the total time duration from initial genetic testing being drawn (at which time the patient was essentially asymptomatic) to being seen in clinic by a pediatric oncologist was only around 3 weeks. Prompt repeat testing and diagnostic evaluation enabled rapid initiation of appropriate oncologic care for this child, likely improving long‐term outcomes.

## 4. Conclusion

Genetic testing in the context of neurodevelopmental disorder or autism evaluation may occasionally reveal incidental findings with urgent medical implications. In this case, CMA uncovered evidence of hypodiploidy, leading to a timely and life‐saving diagnosis of acute leukemia. Pediatric neurologists and other ordering providers should be aware of the possibility of such incidental findings and act swiftly in coordinating care and follow‐up testing.

## Funding

This research received no external funding.

## Ethics Statement

This case report was conducted in accordance with institutional ethical standards and the principles of the Declaration of Helsinki. Institutional Review Board approval was not required for publication of a single retrospective case report in accordance with institutional policy.

## Consent

Informed consent for publication of this case report and any accompanying clinical details was obtained from the patient’s parent/legal guardian.

## Conflicts of Interest

The author declares no conflicts of interest.

## Supporting Information

Additional supporting information can be found online in the Supporting Information section.

## Supporting information


**Supporting Information** CARE Guideline Statement: This case report was prepared in accordance with the CARE Guidelines.

## Data Availability

The data that support the findings of this study are available on request from the corresponding author. The data are not publicly available due to privacy or ethical restrictions.
