# Supplementary material for: Incidental Detection of Acute Leukemia During Genetic Evaluation of Neurodevelopmental Disorder in a Pediatric Clinic: A Case Report
Source: Case Rep Pediatr. 2026 Jul 15;2026:1527918. doi: 10.1155/crpe/1527918 (PMC13373520; doi:10.1155/crpe/1527918)
Supplement: Supplementary file 1 — Supporting Information CARE Guideline Statement: This case report was prepared in accordance with the CARE Guidelines. [file CRPE-2026-1527918-s001.pdf]

CARE case report guidelines

CARE Checklist of information to include when writing a case report

(cc) BY-NC-ND

Topic	Item	Checklist item description	Reported on Line
Title	1	The diagnosis or intervention of primary focus followed by the words "case report"	p3, line 3
Key Words	2	2 to 5 key words that identify diagnoses or interventions in this case report, including "case report"	p4, line 2
Abstract (no references)	3a	Introduction: What is unique about this case and what does it add to the scientific literature?	p3, line 17
	3b	Main symptoms and/or important clinical findings	p3, line 11
	3c	The main diagnoses, therapeutic interventions, and outcomes	p3, line 13
	3d	Conclusion—What is the main "take-away" lesson(s) from this case?	p3, line 17
Introduction	4	One or two paragraphs summarizing why this case is unique (**may include references**)	p4, line 9
Patient Information	5a	De-identified patient specific information.	p4, line 14
	5b	Primary concerns and symptoms of the patient.	p4, line 14
	5c	Medical, family, and psycho-social history including relevant genetic information	p4, line 14
	5d	Relevant past interventions with outcomes	N/A
Clinical Findings	6	Describe significant physical examination (PE) and important clinical findings.	p4, line 20
Timeline	7	Historical and current information from this episode of care organized as a timeline	p5, case presentation
Diagnostic Assessment	8a	Diagnostic testing (such as PE, laboratory testing, imaging, surveys).	p4, line 17, 21, p5 line 8
	8b	Diagnostic challenges (such as access to testing, financial, or cultural)	N/A
	8c	Diagnosis (including other diagnoses considered)	p5, line 7
	8d	Prognosis (such as staging in oncology) where applicable	p5, line 22
Therapeutic Intervention	9a	Types of therapeutic intervention (such as pharmacologic, surgical, preventive, self-care)	p5, line 10
	9b	Administration of therapeutic intervention (such as dosage, strength, duration)	N/A
	9c	Changes in therapeutic intervention (with rationale)	N/A
Follow-up and Outcomes	10a	Clinician and patient-assessed outcomes (if available)	p5, line 10
	10b	Important follow-up diagnostic and other test results	p5, line 14
	10c	Intervention adherence and tolerability (How was this assessed?)	N/A
	10d	Adverse and unanticipated events	p5, line 1
Discussion	11a	A scientific discussion of the strengths AND limitations associated with this case report	discussion
	11b	Discussion of the relevant medical literature **with references**.	discussion, references
	11c	The scientific rationale for any conclusions (including assessment of possible causes)	p5, discussion
	11d	The primary "take-away" lessons of this case report (without references) in a one paragraph conclusion	p7, line 9
Patient Perspective	12	The patient should share their perspective in one to two paragraphs on the treatment(s) they received	N/A
Informed Consent	13	Did the patient give informed consent? Please provide if requested	**Yes** ☑ **No** ☐
